# Automatic classification of normal and abnormal cell division using deep learning

**DOI:** 10.1038/s41598-024-64834-7

**Published:** 2024-06-20

**Authors:** Pablo Delgado-Rodriguez, Rodrigo Morales Sánchez, Elouan Rouméas-Noël, François Paris, Arrate Munoz-Barrutia

**Affiliations:** 1 Universidad Carlos III de Madrid, Leganes, Spain; 2 Centre Régional de Recherche en Cancérologie et Immunologie Intégré Nantes-Angers, Nantes, France; 3 Institut de Cancérologie de L’Ouest, Saint-Herblain, France

**Keywords:** Cancer, Computational biology and bioinformatics, Mathematics and computing

## Abstract

In recent years, there has been a surge in the development of methods for cell segmentation and tracking, with initiatives like the Cell Tracking Challenge driving progress in the field. Most studies focus on regular cell population videos in which cells are segmented and followed, and parental relationships annotated. However, DNA damage induced by genotoxic drugs or ionizing radiation produces additional abnormal events since it leads to behaviors like abnormal cell divisions (resulting in a number of daughters different from two) and cell death. With this in mind, we developed an automatic mitosis classifier to categorize small mitosis image sequences centered around one cell as “Normal” or “Abnormal.” These mitosis sequences were extracted from videos of cell populations exposed to varying levels of radiation that affect the cell cycle’s development. We explored several deep-learning architectures and found that a network with a ResNet50 backbone and including a Long Short-Term Memory (LSTM) layer produced the best results (mean F1-score: 0.93 ± 0.06). In the future, we plan to integrate this classifier with cell segmentation and tracking to build phylogenetic trees of the population after genomic stress.

## Introduction

Cell segmentation and tracking have gained increased attention in recent years due to new and complex data involving large cell populations and diverse behaviors. Initiatives such as the Cell Tracking Challenge (CTC)^1^ have brought together researchers to benchmark and advance the field. While manual methods for cell tracking (such as the one proposed in^2^ for glioblastoma populations) can provide valuable insights into behavior, it is necessary to develop automatic methods to extrapolate this to new videos. Tracking typically involves storing the cells’ location at each timestep and its relationship with cells in previous and subsequent frames, including whether the current cell is a continuation of a previous cell or a descendant from cell division. These parameters are commonly obtained using current cell tracking algorithms, as demonstrated in the CTC Benchmarking initiative^1,3^. However, different types of videos can give rise to additional variables. For example, in the analysis of videos that capture the cell response after genomic stress, such as radiation in therapeutical doses. Radiotherapy is widely used to compromise tumor cells and inhibit their growth^4,5^, causing DNA damage that leads to unusual behaviors during mitosis, when un- or mis-repaired. In such cases, it is essential to classify each mitosis in the video as normal or abnormal to measure the disruptive effects in cell toxicity. In this work, we utilized videos of glioblastoma cell populations exposed to varying radiation levels to train and test our proposed algorithm to create a generalizable method that can be applied to different videos from future experiments.

**Figure 1:**
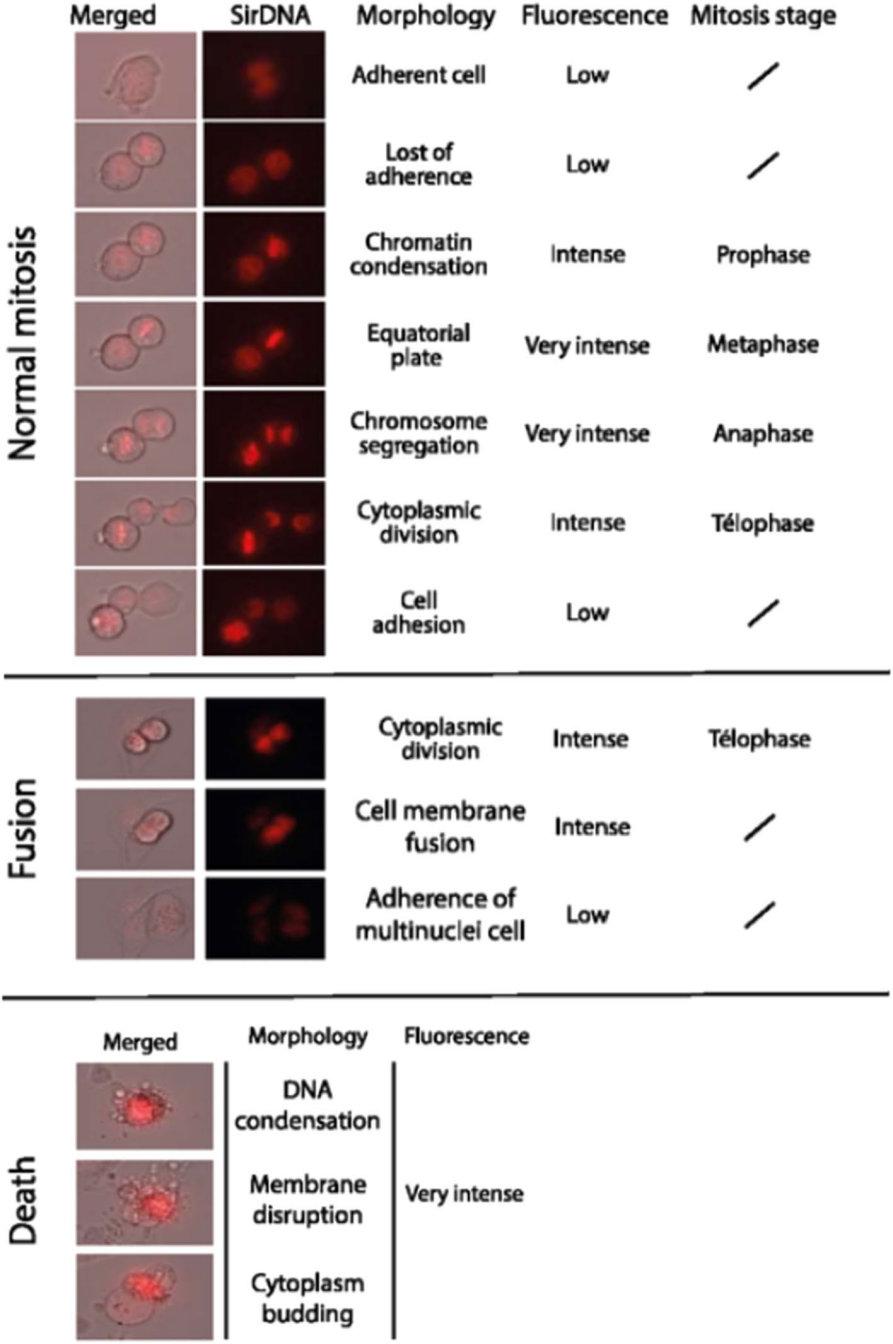
Illustration of normal mitosis, fusion, and cell death. Representative merged images (phase contrast microscopy and SirDNA); SirDNA fluorescence microscopy images; description of the cell morphology, the fluorescence level and for the normal mitoses and the fusion, the mitosis stage. The mitosis stages show how the cell starts adhered to the plate, it detaches, the chromatin gets condensed forming a plate in the middle of the cell body, then the chromosomes are pulled to opposite borders of the cell and the cytoplasm divides. After this, both daughter cells attach again to the plate. On the contrary, in fusion, after the cytoplasm has divided it remerges again into a single cell that then attaches back to the plate. In cell death, on the other hand, the genetic material condenses, the membrane breaks and bubbles are formed.

Figure 1 illustrates the steps of normal mitosis, abnormal mitosis resulting from cell fusion, and cell death. Normal mitosis involves two arising cells re-adhering to the plate, while cell fusion results in a single multinucleated cell. Videos were recorded using a contrast phase channel to show cell shape and a far-red fluorescent channel to visualize nuclei after Sir-DNA counterstaining. To automatically analyze them, it is necessary to identify whether a given cell division (mitosis) is normal or abnormal, which could indicate that ionizing through DNA damage has perturbed the cell cycle. For non-experts, distinguishing between normal and abnormal mitosis can be challenging due to the various ways cell division can fail.

Most automated methods in the literature on mitosis analysis focus on detecting normal mitosis in 2D images, primarily in histology slices, to assess tumor growth^6–10^. Other studies address the evolution of this process through time^11–13^ to track cell division in time-lapse videos, either classifying image crops as mitosis or non-mitosis^14^ or applying classification to single-cell images^15^. These studies do not consider other cell behaviors besides normal mitosis. Besides, cell division occurs over a series of time steps, and the distinction between mitosis types is only apparent when considering temporal progression, which is why we propose to analyze short time sequences centered around dividing cells. Deep Learning (DL) algorithms have proven effective for classifying single-cell 2D images^16^, so we explored some of these models to work with our mitosis sequences and optimized an architecture with the best hyperparameters to accurately perform the classification. It could be later integrated into a segmentation and tracking pipeline to characterize the entire population.

## Materials and methods

### Cell culture and image acquisition

We used human glioblastoma (U251) and prostate adenocarcinoma (PC3) cell lines purchased at the American Type Culture Collection (ATCC). All biological experiments, including cell culture, irradiation and videomicroscopy were approved by an institutional committee, following the guidelines and regulations of the Institut National de la Santé et de la Recherche Médicale (Inserm) and Nantes University. Informed consent has been obtained from all authors and their legal institution(s) for all subjects covering the entire manuscript.

The cells were cultured in DMEM supplemented with 10% SVF, 1% penicillin–streptomycin, and 1% L-glycine under standard conditions at 37 °C/5% CO2. Cell irradiation was performed with a CP-160 X-ray irradiator (Faxitron, Lincolnshire, Illinois, USA) at an accelerating voltage of 160 kV, dose of 1.3 Gy/min. For each radiation level (0, 4 and 10 Gy), we prepared two different culture wells in which cells were plated at a density of 7500 cells/cm^2^. These wells were placed on a microscope equipped with an incubator for long-term imaging. We captured images of 10 to 20 cells at baseline using a 20X magnification lens, at three separate locations within each well. This imaging was conducted at 10 min intervals over a period of 5 days with a Nikon Ti inverted optical microscope (Nikon, Minato-ku, Tokyo, Japan). Both brightfield and far-red fluorescence signals were acquired (2 channels), to observe cell morphology and nuclear labeling by SiR-Hoechst (SiR-DNA, Spirochrome).

### Image processing

**Figure 2:**
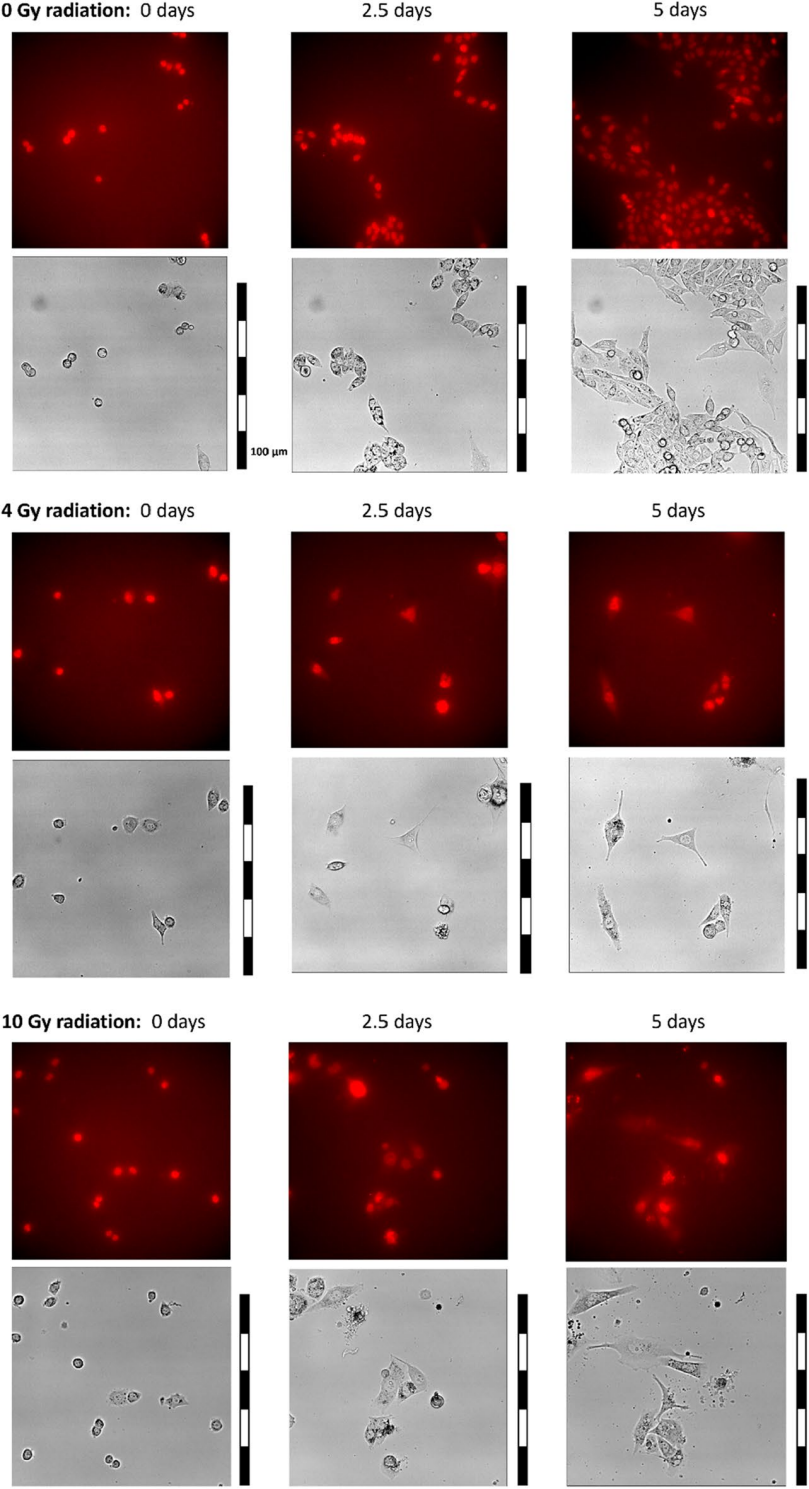
Example of cell population videos with different levels of radiation. Both channels are shown (fluorescence and phase contrast, in this order). Three timesteps: 0, 2.5, and 5 days. Top. No radiation; Middle. 4 Gy radiation. Bottom. 10 Gy radiation. Cells present more abhorrent behaviors as radiation increases, also reducing their replications.

This initially obtained data consisted of 2D videos showing cell populations under different radiation levels, each of them composed of 721 frames of 1024 × 1024 pixels. Figure 2 shows three of these videos at 0, 2.5, and 5 days for different radiation levels (0 Gy, 4 Gy, and 10 Gy). It can be observed that the highest number of mitoses, producing a larger population, occurs for 0 Gy, while an increase in radiation difficults the normal progress of the cell cycle.

**Figure 3:**
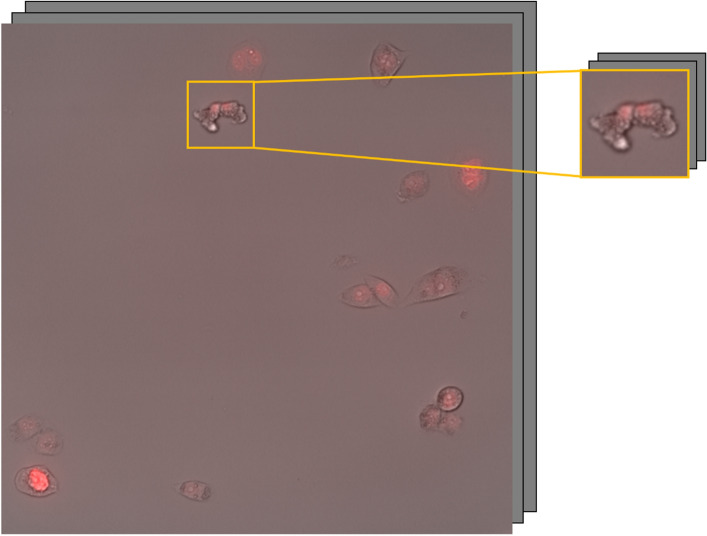
Example of a manual mitosis sequence extraction from a complete cell population video.

From these complete videos, smaller image sequences were manually extracted, centered around a mitosis event (Fig. 3). They were created by drawing a square around the mitosis event and extending it for 12 frames ensuring that each frame included both daughters. This corresponds to a period of two hours. The resulting sequence was stored as a separate image, preserving both channels, with frame sizes ranging from 50 × 50 to 110 × 110 pixels. The sequence started just before cell division, when the parent cell had already detached from the plate and became round, and finished when both daughters had attached to the plate. This allowed the entire division process to be observed while maintaining a manageable data size. 47 sequences of each class (normal and abnormal mitosis) were stored as test data, while the remaining sequences were used for training the algorithms. We removed the sequences that were ambiguous for human classification to train our models to replicate human behavior. 146 abnormal and 317 normal sequences were used for the training set, while 47 normal and 47 abnormal were the test set. During training, we aimed to use as many images as possible, and class imbalance was addressed by weighting each class based on its proportion to the total.

**Figure 4:**
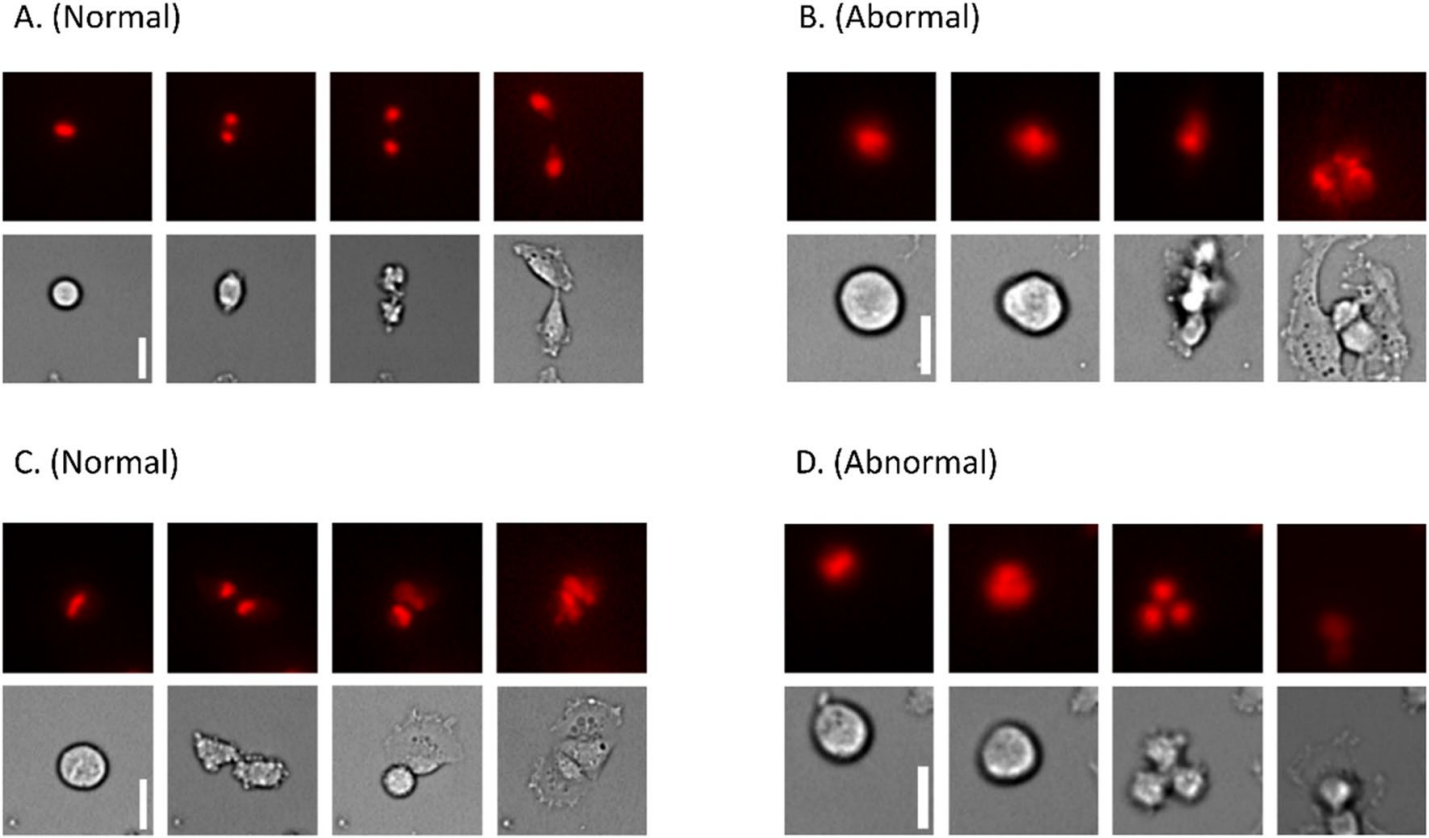
Normal vs. abnormal mitosis examples, the white scale bars represent 10 µm. Normal mitosis typically ends with two differentiated cells adhered to the plate, while abnormal mitosis may result in abnormal behaviors such as forming three cells. The image displays four frames selected from multiple mitosis substacks (original substacks consist of 12 frames, covering a period of two hours). (**A**,**C**) Normal mitoses. (**B**,**D**) Abnormal mitoses. 4 frames are shown for each of the short videos, including the initial state, two significant middle states and the final state.

Figure 4 shows some frames from mitosis sequences. It can be observed how during normal mitosis the cell becomes round, condensing the chromosomes in the middle region. Then, it divides its genetic material, which migrates to each daughter cell while the cytoplasm divides. At the end of the process, two new cells are created, which can be seen in sequences Fig. 4A,C. On the other hand, abnormal mitosis can show several abhorrent behaviors. In Fig. 4B, the daughter cells become intermingled and end up forming a composed cell, while in sequence Fig. 4D, the division gives rise to three cells instead of two.

Multiple DL architectures were trained on the sequences to classify new samples as normal or abnormal mitosis. The temporal dimension of the samples is a unique aspect of this problem. One approach could be to treat each sequence as a 3D image and use 3D convolutions, but instead, we used a Long Short-Term Memory (LSTM) layer to consider the temporal relationship between frames and produce an output summarizing the video's characteristics.

**Figure 5:**
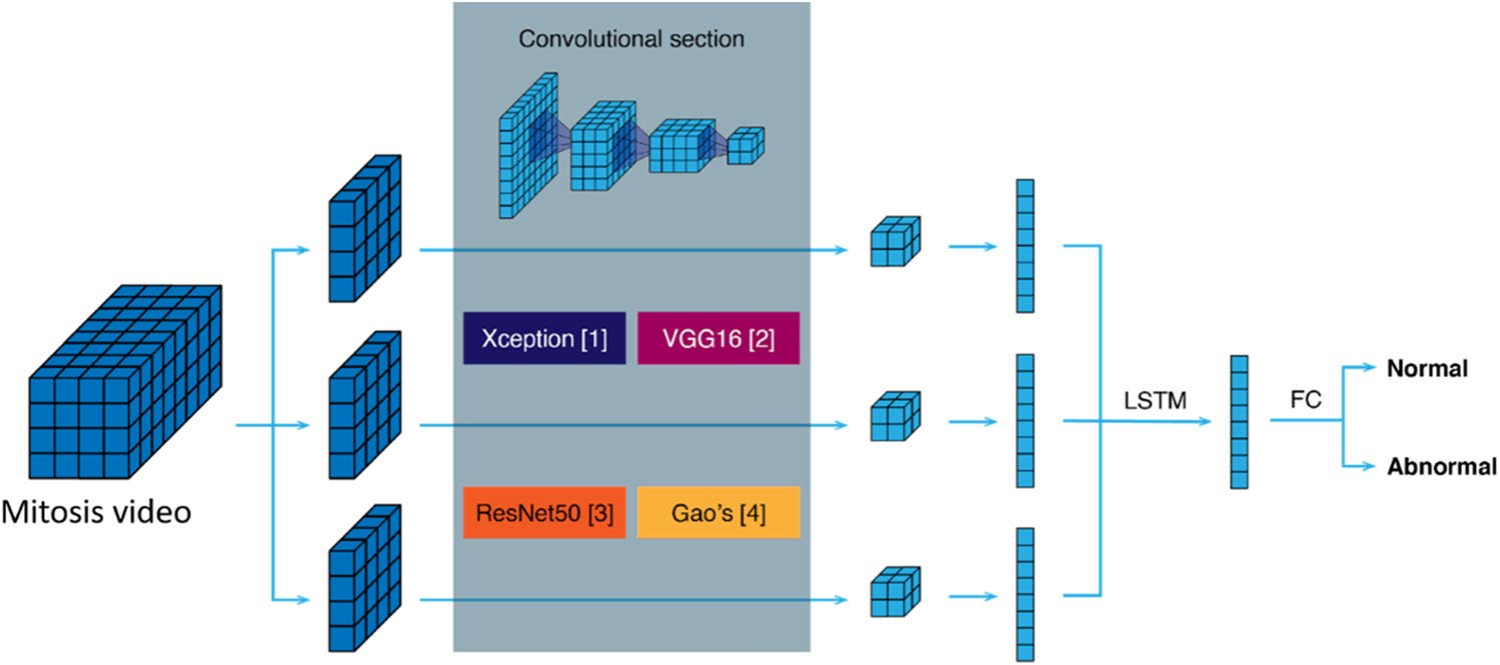
Diagram of the network architectures. For each mitosis video frame, it goes through the same set of convolutional layers (Xception, VGG16, Resnet50 or Gao’s architectures) and is reduced to a feature vector. These vectors are then input to an LSTM layer to capture the temporal relationships and produce an output vector. The output vector goes through FC layers and finally yields a normal or abnormal classification.

The structure of the pipeline is shown in Fig. 5. A convolutional section topped the network and was applied to each frame using a "time distributed" layer, with the same set of weights for all frames. The output from each frame was vectorized and fed into a Long Short-Term Memory (LSTM) layer to produce a single summary of the video's information. Finally, FC layers and a SoftMax layer were used to obtain the classification. We tested the next structures for the convolutional section of the network: VGG16^17^, which is structured based on the use of 3 × 3 convolutions, smaller than its predecessors, as well as 1 × 1 convolutions to introduce more non-linearity; Xception^18^ based on the Inception network^19^ and using depthwise separable convolutions; ResNet50^20^, using residual layers that include skip connections, and Gao’s model, from^15^, a series of layers created for cell classification.

To enhance the algorithms' ability to handle diverse data and prevent overfitting, several data augmentation operations were applied to each sequence. This includes random vertical and horizontal flip, random rotation from 0° to 90°, random noise addition (noise following a uniform distribution), random elastic deformation of a 3 × 3 grid of nodes following a uniform distribution, interpolation of the rest of the image.

Multiple hyperparameter configurations were tested for each proposed architecture to find the optimal one and achieve the best classification. Each network was trained with each configuration 10 times, yielding a mean and standard deviation of their performance for a more reliable evaluation. The training was capped at 500 epochs and would stop if validation accuracy did not significantly improve in the last 100 epochs. The validation accuracy was obtained by evaluating the model on 43% of the randomly split training data, not used to optimize the network weights. This high validation proportion was chosen to ensure the model's generalizability to new data. After each training, the weights with the highest validation accuracy were retained as “best weights”. A key parameter to analyze was the impact of starting from a network pretrained on ImageNet, compared to training from scratch. The training was conducted using Google Colab pro^21^, with GPU access to accelerate the computations.

On the other hand, extra trainings were performed to check the relevance of these LSTM methods against an alternative, more traditional algorithm that did not involve temporal analysis. Once the best-performing LSTM architecture and hyperparameter configuration had been determined in the previous step, a 3D version of the backbone corresponding to that architecture was trained, in this case taking the mitosis sequences as 3D images. This network was directly applied to each mitosis sequence, without the use of a time-distributed layer or an LSTM layer, yielding a classification output.

## Results and discussion

For each hyperparameter configuration, we performed ten identical trainings, storing the best validation accuracy, precision, recall, and F1-score, which enabled us to obtain statistical measures of the performance of each configuration. The mean and standard deviation from these ten runs are displayed in Table 1. The best-performing model on validation from each architecture was selected and the ten trained models corresponding to that setup were evaluated on the test set to determine their performance on unseen data. These results can be seen in Table 2. The F1-score results, used as an overall score, always exceeded 0.79, indicating all the algorithms performed well, with some exhibiting particularly reliable behavior.

**Table 1:** Validation results for various architecture and hyperparameter configurations are displayed.

Architecture	Varying hyperparameters			Results on the validation set			
	Pretrained	Initial learning rate	Batch size	Mean val. accuracy	Mean val. precision	Mean val. Recall	Mean val. F1-score
Xception	Yes	0.001/ 0.00001	10	0.9 ± 0.03	0.82 ± 0.07	0.76 ± 0.15	0.77 ± 0.09
Xception	No	0.001	10	0.92 ± 0.02	0.89 ± 0.07	0.77 ± 0.07	0.82 ± 0.05
**Xception**	**No**	**0.0001**	**10**	**0.96 ± 0.03**	**0.98 ± 0.02**	**0.86 ± 0.12**	**0.91 ± 0.07**
Xception	No	0.00001	10	0.93 ± 0.04	0.88 ± 0.07	0.85 ± 0.07	0.86 ± 0.08
Xception	No	0.0001	20	0.94 ± 0.05	0.94 ± 0.06	0.8 ± 0.18	0.86 ± 0.13
VGG16	Yes	0.001/ 0.00001	10	0.94 ± 0.02	0.92 ± 0.06	0.82 ± 0.13	0.86 ± 0.07
VGG16	No	0.001	10	0.75 ± 0	0 ± 0	0 ± 0	0 ± 0
VGG16	Yes	0.0001/ 0.00001	10	0.75 ± 0	0 ± 0	0 ± 0	0 ± 0
**VGG16**	**Yes**	**0.00001/ 0.000001**	**10**	**0.94 ± 0.02**	**0.91 ± 0.07**	**0.84 ± 0.11**	**0.86 ± 0.05**
VGG16	Yes	0.00001/ 0.000001	20	0.93 ± 0.04	0.9 ± 0.1	0.83 ± 0.1	0.86 ± 0.09
**ResNet50**	**Yes**	**0.001/ 0.00001**	**10**	**0.95 ± 0.04**	**0.89 ± 0.1**	**0.91 ± 0.1**	**0.9 ± 0.09**
ResNet50	No	0.001	10	0.82 ± 0.04	0.66 ± 0.1	0.53 ± 0.14	0.58 ± 0.11
ResNet50	Yes	0.0001/ 0.00001	10	0.94 ± 0.05	0.93 ± 0.08	0.81 ± 0.17	0.86 ± 0.13
ResNet50	Yes	0.00001/ 0.000001	10	0.95 ± 0.04	0.91 ± 0.1	0.89 ± 0.09	0.9 ± 0.09
ResNet50	Yes	0.001/ 0.00001	20	0.85 ± 0.05	0.8 ± 0.11	0.51 ± 0.15	0.62 ± 0.14
**Gao**	**No**	**0.001**	**10**	**0.86 ± 0.05**	**0.74 ± 0.1**	**0.69 ± 0.2**	**0.7 ± 0.15**
Gao	No	0.0001	10	0.83 ± 0.07	0.65 ± 0.13	0.75 ± 0.11	0.69 ± 0.1
Gao	No	0.00001	10	0.72 ± 0.03	0.33 ± 0.18	0.31 ± 0.2	0.31 ± 0.18
Gao	No	0.001	20	0.84 ± 0.04	0.66 ± 0.24	0.58 ± 0.21	0.61 ± 0.21

Different colors (blue, purple, orange, and yellow) indicate different architectures, with the best-performing model for each highlighted in bold and with a thicker border. Each training run was repeated 10 times, and the best validation accuracy, precision, recall, and F1-score were averaged to produce a mean ± standard deviation value for each. The best-performing models were selected primarily based on the F1-score.

**Table 2:** Test scores for the best-performing model of each architecture. The model with the highest mean F1-score and lowest standard deviation (resnet50) is highlighted in bold.

Architecture	Varying hyperparameters			Results on the test set			
	Pretrained	Initial lr	Batch size	Mean accuracy	Mean precision	Mean Recall	Mean F1-score
Xception	No	0.0001	10	0.94 ± 0.07	1 ± 0.01	0.88 ± 0.14	0.93 ± 0.1
VGG16	Yes	0.00001/ 0.000001	10	0.89 ± 0.05	0.95 ± 0.03	0.83 ± 0.11	0.88 ± 0.06
**ResNet50**	**Yes**	**0.001/ 0.00001**	**10**	**0.94 ± 0.05**	**0.97 ± 0.03**	**0.9 ± 0.09**	**0.93 ± 0.06**
Gao	No	0.001	10	0.82 ± 0.08	0.93 ± 0.03	0.71 ± 0.16	0.79 ± 0.12

The approach presented here aims to automate the analysis of videos of cell populations subjected to toxicity. Tracking and segmenting these cells can provide valuable information on their behavior under different levels of toxicity; however, cells can exhibit unusual behaviors in these cases, particularly unstable cell division, which can be challenging for the human eye to evaluate quickly. The proposed network can accurately classify mitoses and addresses the need for an automatic tool to tackle this specific task.

One of the objectives was to assess the impact of using a pretrained network on mitosis classification results, even if the pretraining dataset (ImageNet) contained images unrelated to microscopy. Only networks with widely used backbones (ResNet, VGG, or Xception) could be pretrained. As expected, these versions of VGG16 and ResNet50 performed better. Nevertheless, Xception showed better average results when trained from scratch. This could be because this architecture is more capable of working directly with our images.

**Figure 6:**
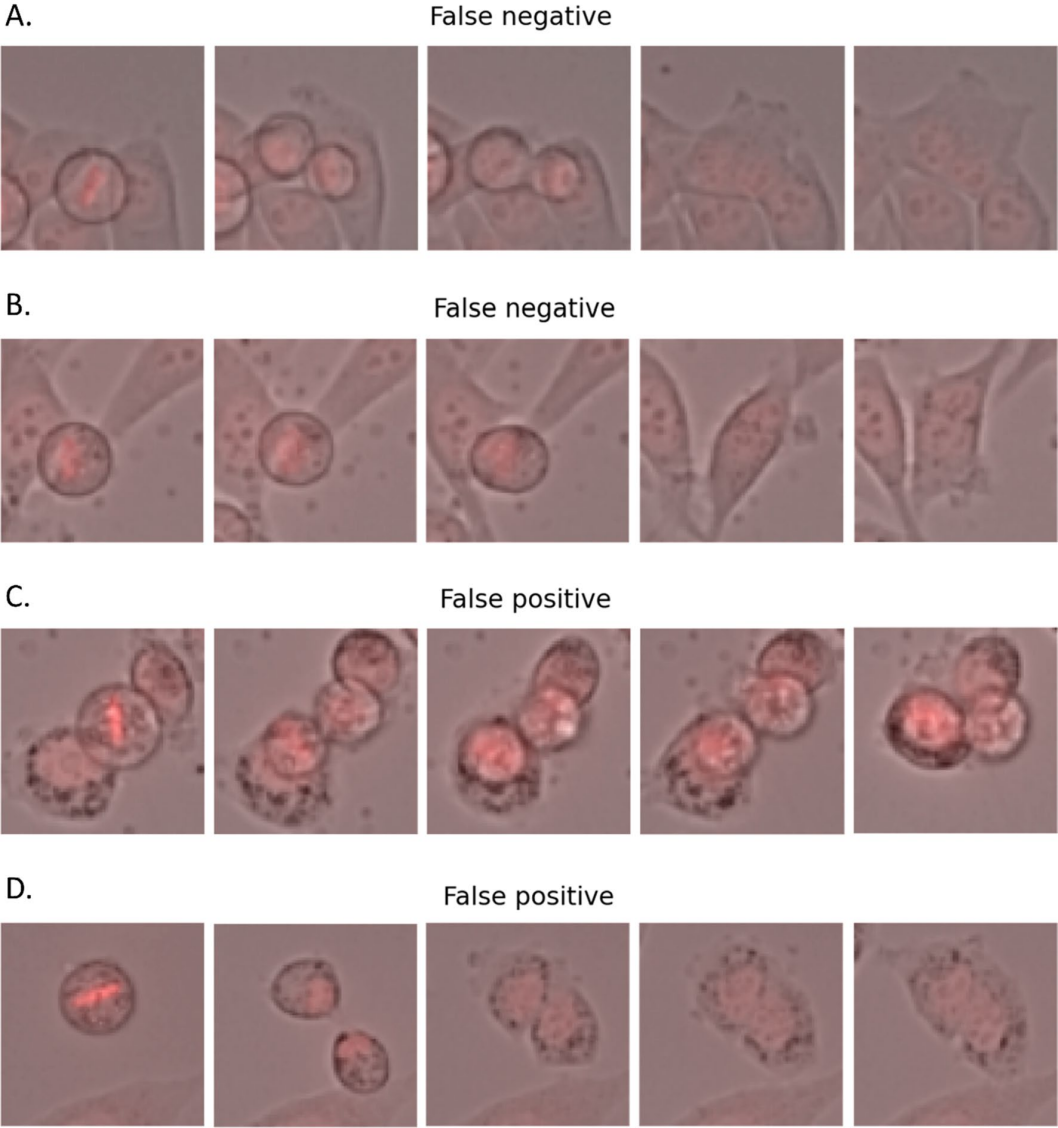
Examples of false negatives (abnormal mitosis classified as normal) and false positives (normal mitosis classified as abnormal) from the ResNet50 model applied to test data. Each example displays 4 of the 12 frames in each mitosis sample. In (**A**), a cell clearly divides completely and then reunites, making the mitosis abnormal, but the algorithm might mistake the initial division as normal. In (**B**), the cell tries to divide but fails, leading the algorithm to mistake a different cell in the image as a second daughter and classify the mitosis as normal. In (**C**), the cell divides correctly, but a different cell appearing in the image might confuse the network into misclassifying the mitosis as abnormal. In (**D**), both daughter cells end up separate, but the algorithm might mistake them as one cell, leading to classification failure.

Regarding the initial learning rate of the optimizer, both Xception and VGG16 architectures performed best on the validation set with a lower value. VGG16 offered no true positives for both the configuration trained from scratch with 0.001 as initial learning rate and the pretrained configuration for an initial rate of 0.0001. This behavior was mitigated by decreasing the initial learning rate to 0.00001 for a pretrained configuration, which led VGG16 to achieve optimal results. ResNet50 offered higher performance for the pretrained configuration without using very low initial learning rates. On the other hand, the model based on Gao's architecture performed better with a higher learning rate of 0.001, as it was not pretrained and needed larger steps to find a minimum. Changing the batch size did not have a significant impact.

the best versions of ResNet50 (pretrained) and Xception (trained from scratch) outperformed all other models, with a mean F1-score of 0.93 on test data. ResNet50 had a lower standard deviation, making it the best model overall after considering high individual scores for accuracy, precision, and recall. Surprisingly, a trained-from-scratch Xception model performed comparably to a ResNet50 model with prior knowledge. The chosen top-performing algorithm, ResNet50, provides accurate results for the classification problem and can support human experts in the classification task. Figure 6 shows examples of mitoses misclassified by the ResNet50 model. These errors could be attributed to several factors, as the network can be easily misled by events like overlapping cells, crowded surroundings, poor contrast in some videos, and instances where the daughter cells do not have the time to re-attach to the surface during the video (Fig. 6D). It can be concluded that the algorithmic pipeline is particularly adept at identifying mitosis as normal or abnormal when cells are not too crowded together and in the cases (most of them) where a selection of 12 frames covers the whole mitotic process.

As mentioned in the previous section, a 3D version of ResNet50 was trained using the same hyperparameter configuration as the best-performing model, in order to compare a non-temporal model with our proposed algorithm. This 3DResNet architecture was extracted from the GitHub repository^22^ with some slight modifications applied for input and output formatting. The results for both training from scratch and fine-tuning a model pretrained on the Kinetics-700 database can be seen in Table 3, compared to the ones obtained by our proposed ResNet50 LSTM network. As the results show, the same parameters that were used during the training of the temporal analysis models yield as a result in these 3D models an insufficient understanding of the data. The recall is very high with a low precision, which implies that most of the input data is being classified as abnormal mitoses. The network, thus, shows difficulty in differentiating between normal and abnormal mitoses when they are presented as a single block instead of a series of steps, and prefers to mark almost all inputs as abnormal. This indicates the relevance of our proposed architecture, that is able to extract the pertinent information from the videos and provide adequate results.

**Table 3:** Comparison of mean test scores by the best ResNet50 backbone LSTM model against a 3DResNet model without temporal analysis (both pretrained and trained from scratch).

Architecture	Mean accuracy	Mean precision	Mean recall	Mean F1-score
Best ResNet50 LSTM	0.94	0.97	0.9	0.93
3D ResNet50 (from scratch)	0.479	0.489	0.957	0.647
3D ResNet50 (pretrained)	0.468	0.483	0.936	0.638

Lastly, the best-performing ResNet50 LSTM network was applied to a series of additional mitosis sequences manually extracted from three extra PC3 cell population videos to evaluate its performance on new prostate cancer data that was not present during the whole training and validation process. This smaller set was composed of 24 mitoses: 4 abnormal and 20 normal. The results are shown in Table 3. The high recall shows that the proportion of true positives over all the abnormal images is good enough to properly predict most of the abnormal mitoses. Although the precision is lower than the one shown for the previous, larger test dataset, this is partly due to the fact that this new set of data includes much fewer abnormal mitoses, so the number of true positives cannot be too high. Nevertheless, the overall accuracy is higher than 0.8, which means that most of the videos are correctly classified. Despite the scores being somehow lower than for the previous test set, this new application shows an example of how the algorithm could be applied over a single new set of videos, even if its classes are unbalanced (Table 4).

**Table 4:** Scores for the best-performing ResNet50 LSTM model on 24 new mitosis sequences from 3 additional PC3 cell population videos. The dataset was composed of 20 normal and 4 abnormal mitoses.

Architecture	Accuracy	Precision	Recall	F1-score
Best ResNet50 LSTM	0.834	0.500	0.750	0.600

## Conclusion

Our goal was to simplify the task of analyzing cell mitosis by developing an automatic method for classifying mitotic events as normal or abnormal. After evaluating different configurations, our results demonstrated that a neural network with a ResNet50 backbone and an LSTM layer accurately classified small mitosis video sequences, achieving a mean F1-score of 0.93 ± 0.06. Interestingly, pretraining on ImageNet may not be essential for this type of image analysis, as a similar score was obtained using an Xception backbone without pretraining, which suggests that this architecture might be particularly effective for analyzing microscopy data.

This model has been compared with an equivalent 3D network that does not take into account any temporal information and takes the mitosis sequences as 3D arrays. The results show a much less accurate performance in this case, which supports the need for our proposed LSTM model.

Given the growing importance of segmentation and tracking algorithms to characterize the cellular behavior within specific environments, the classifier presented here can streamline a tedious task and be integrated into a larger workflow, such as a mitosis detection system, to identify the locations of normal and abnormal mitoses over time. In the future, this approach could enable a comprehensive understanding of the cell population's behavior under radiation or other genomic stresses. It could be used to observe the effects of these treatments on common 2D cultures, such as glioblastoma shown in this present work. Additionally, it could potentially be expanded to discriminate the different types of abnormal mitosis, including fusion, non-dichotomic division (in three or more cells) or loss of genomic materials in micronuclei.

Furthermore, the proposed method can be applied to microscopy images obtained from different studies investigating the effects of various toxic chemical compounds on normal cell cycle development. The key aspect is that this algorithm can be retrained with the appropriate set of images, using fine-tuning to allow researchers to automate mitosis classification tasks and characterize the development of cell populations exposed to other forms of toxicity.

## Data Availability

A version of the code has been made available as a Google Colab notebook in the style of ZeroCostDL4Mic^23^ (https://github.com/HenriquesLab/ZeroCostDL4Mic), aiming to facilitate its reusability by not-programming users. This notebook allows training the different architectures on a set of mitosis videos (12 frames each) specified by the user and is available at^24^ (https://github.com/pdelgado248/mitosis-classification/tree/main). This code uses a version of the github video generator repository^25^ (https://github.com/metal3d/keras-video-generators). Besides, the train and test mitosis videos, the weights stored from the main trainings, the additional 24 mitosis sequences and the 3DResNet training weights can be found in Zenodo, at^26^ (10.5281/zenodo.7788748).
